# Differential Association of Niemann-Pick C1 Gene Polymorphisms with Maternal Prepregnancy Overweight and Gestational Diabetes

**DOI:** 10.15436/2376-0494.15.007

**Published:** 2015-02-27

**Authors:** William S. Garver, Lesley de la Torre, Matthew C. Brennan, Li Luo, David Jelinek, Joseph J. Castillo, David Meyre, Robert A. Orlando, Randall A. Heidenreich, William F. Rayburn

**Affiliations:** 1 Department of Biochemistry and Molecular Biology, University of New Mexico Health Sciences Center, Albuquerque, New Mexico, USA; 2 Department of Obstetrics and Gynecology, University of New Mexico Health Sciences Center, Albuquerque, New Mexico, USA; 3 Department of Internal Medicine, University of New Mexico Health Sciences Center, Albuquerque, New Mexico, USA; 4 Department of Clinical Epidemiology and Biostatistics, McMaster University, Hamilton, Canada; 5 Department of Pediatrics, University of New Mexico School of Medicine, Albuquerque, New Mexico, USA

**Keywords:** Gestational diabetes, Niemann-Pick C1, Obesity, Obstetrics, Overweight, Polymorphism

## Abstract

A genome-wide association study (GWAS) and subsequent replication studies in diverse ethnic groups indicate that common Niemann-Pick C1 gene (*NPC1*) polymorphisms are associated with morbid-adult obesity or diabetes independent of body weight. The objectives for this prospective cross-sectional study were to determine allele frequencies for *NPC1* polymorphisms (644A>G, 1926C>G, 2572A>G, and 3797G>A) and association with metabolic disease phenotypes in an ethnically diverse New Mexican obstetric population. Allele frequencies for 1926C>G, 2572A>G, and 3797G>A were significantly different between race/ethnic groups (non-Hispanic white, Hispanic, and Native American). The results also indicated a significant pairwise linkage-disequilibrium between each of the four *NPC1* polymorphisms in race/ethnic groups. Moreover, the derived and major allele for 1926C>G was associated (OR 2.11, 95% CI 1.10–3.96, P = 0.022) with increased risk for maternal prepregnancy overweight (BMI 25.0–29.9kg/m^2^) while the ancestral and major allele for 2572A>G was associated (OR 4.68, 95% CI 1.23–17.8, P = 0.024) with increased risk for gestational diabetes in non-Hispanic whites, but not Hispanics or Native Americans. In summary, this is the first transferability study to investigate common *NPC1* polymorphisms in a multiethnic population and demonstrate a differential association with increased risk for maternal prepregnancy overweight and gestational diabetes.

## Introduction

The most recent National Health and Nutrition Examination Survey (NHANES) indicates that approximately two-thirds (59.5%) of reproductive age (20 to 39 years of age) women are overweight (BMI ≥ 25kg/m^2^) and approximately one-third (34.0%) of reproductive age women are obese (BMI ≥ 30kg/m^2^) in the United States^[1]^. Maternal overweight and obesity are believed to be responsible for perpetuating the current epidemic of obesity in children and adults through *in utero* reprogramming and epigenetic mechanisms^[2]^. Furthermore, maternal overweight and obesity or excessive gestational weight gain is associated with a greater risk for obstetric complications, including gestational diabetes, pre-eclampsia, fetal macrosomia, labor induction, and cesarean delivery^[3]^. Studies indicate that maternal overweight, obesity, and gestational diabetes are more prevalent among Hispanics and Native Americans compared with non-Hispanic whites^[4,5]^. These health disparities are of particular importance to New Mexico which has the largest combined population of Hispanics (46.3%) and Native Americans (9.4%) in the United States^[6]^.

More than 60 obesity susceptibility loci have been identified using genome-wide association studies (GWAS), mainly in populations of European ancestry, and studies are currently being performed to determine the frequency and transferability of these variants in different populations around the world^[7,8]^. An early GWAS revealed that the Niemann-Pick C1 gene (*NPC1*) is associated with morbid-adult obesity (≥ 40kg/m^2^) in an European population^[9]^. Subsequent studies performed to determine the transferability of *NPC1* obesity risk alleles have also discovered alleles associated with decreased fasting insulin levels and increased risk for type 2 diabetes independent of body weight in other populations^[10–12]^. These findings were noteworthy since *NPC1* mutations were originally known to be responsible for a rare and fatal autosomal-recessive lipid-storage disorder called *NPC1* disease^[13,14]^. With respect to this disease, the *NPC1* derived and minor allele for 3182T>C encoding the Ile1061Thr residue predisposes to most diagnosed cases of *NPC1* disease in the United States, particularly among Hispanic patients living in the Northern Rio Grande Valley region of New Mexico and Colorado^[14]^. Population studies suggest that this *NPC1* mutation may have resulted from a founder effect originating from Spanish descendants that migrated and settled in this region^[15, 16]^.

The *NPC1* gene encodes a large and complex membrane-spanning protein localized to a novel late endosome-like compartment that transiently interacts with conventional late endosomes and lysosomes involved in endocytosis^[17,18]^. Studies indicate that the NPC1 protein is expressed in all tissues and has a central role in regulating the transport of lipoprotein-derived lipid (cholesterol and fatty acids) from late endosomes and lysosomes to other cellular compartments to maintain cellular, tissue, and whole body lipid homeostasis^[19–21]^. The physiological mechanisms describing how *NPC1* loss-of-function mutations or polymorphisms predispose to either *NPC1* disease or common metabolic diseases (obesity and diabetes) remain undefined.

The objectives of this prospective cross-sectional study are to determine allele frequencies, linkage-disequilibrium structure and transferability of *NPC1* polymorphisms previously reported to be associated with morbid-adult obesity or diabetes independent of body weight, and to determine whether these *NPC1* polymorphisms are associated with metabolic disease phenotypes in an ethnically diverse New Mexican obstetric population.

## Methods

### Subjects

This study was designed as a prospective cross-sectional study. The research protocol was reviewed and approved by an internal review board from the University of New Mexico Human Research Review Committee (UNM HRRC 10-517). Eligibility criteria included all English and Spanish speaking women admitted to the University of New Mexico Hospital (UNMH) Labor and Delivery Unit whom had received prenatal care. Those women whom had not received prenatal care were excluded from participating in the study. The patient’s prenatal record was accessed through an electronic medical record system to obtain data including maternal race/ethnicity, prepregnancy weight and height, and screening for gestational diabetes. The race/ethnicity of patients was self-reported as being either non-Hispanic white, Hispanic, Native American, Asian-American, or African-American. Maternal prepregnancy body mass index (BMI) was measured by professional staff and categorized into the following groups: normal weight (BMI 18.5–24.9kg/m^2^), overweight (BMI 25.0–29.9kg/m^2^), class I obese (BMI 30.0–34.9kg/m^2^), class II obese (BMI 35.0–39.9kg/m^2^), and class III obese (BMI ≥ 40.0 kg/m^2^). After delivery, eligible patients were informed of the study and invited to participate by providing written consent. Blood collected after admission was transferred to the laboratory for *NPC1* genotype and HbA1c analysis. The HbA1c level was determined with whole blood using a validated Siemens Healthcare Diagnostics DCA Vantage HbA1c Analyzer and Siemens-branded DCA 2000+ HbA1c cartridges (Siemens Corp., Tarrytown, NY). The DNA was isolated from 300μL of whole blood using a Blood Core Kit according to the manufacturer (Qiagen Inc., Gentra Puregene, Valencia, CA).

### Genotype Analysis

To perform *NPC1* genotype analysis, an allele-specific oligonucleotide polymerase chain reaction (ASO-PCR) method was employed which permitted detection of polymorphisms using perfectly-matched oligonucleotides that served as primers for amplification. The analysis was performed at four loci within the *NPC1* coding region designated as follows: rs1805081 (644A>G alleles encoding the H215R residues), rs1788799 (1926C>G alleles encoding the I642M residues), rs1805082 (2572A>G alleles encoding the I858V residues), and rs1805084 (3797G>A alleles encoding the R1266Q residues). The first three *NPC1* polymorphisms have been found to be associated with increased risk for either morbid-adult obesity or type 2 diabetes independent of body weight, while the fourth *NPC1* polymorphism has not been found to be associated with either metabolic disease but included as a negative control loci encoding for residues positioned near the carboxyl-terminus of the NPC1 protein. To perform *NPC1* genotype analysis the following reagents were added to a PCR reaction tube: 2μL of 10x Mango buffer (loading dye); 1μL of 10mM dNTPs; 2μL of 50mM MgCl_2_; 0.5μL of 1μU/μL Taq polymerase; 1μL of 10μM primers designed for the ancestral and derived allele; and 11.5μL H_2_O. The combined reagents were added to 1μL of 50 ng/μL isolated patient template DNA. The amplified PCR products were separated using 2.0% agarose gel electrophoresis and visualized with ultraviolet light. The *NPC1* genotype analysis call rate for all polymorphisms was greater than 99%. Human cell-lines possessing the known *NPC1* polymorphisms were purchased from the Coriell Institute for Medical Research (Camden, NJ) and used as negative and positive controls to confirm patient *NPC1* genotype analyses. The resulting genotypes at each loci were categorized into one of three groups: i) homozygous for the ancestral allele, ii) heterozygous for an ancestral and derived allele, or iii) homozygous for the derived allele. The designation and nomenclature used when referring to alleles and encoded residues is in accordance with the National Center for Biotechnology Information (NCBI) and Ensembl Genome (http://useast.ensembl.org/index.html) which use the most recent ancestor for designating ancestral allele for the human genome by sequence alignment between human and five primates (chimpanzee, gorilla, orangutan, macaque, and marmoset). The allele status of each *NPC1* polymorphism in archaic humans (Neanderthal and Denisovan) was also obtained from the Neanderthal genome and the high-coverage Denisovan genome using UCSC Genome (https://genome.ucsc.edu/). This information of allele status in primates and archaic humans is helpful to understand whether a specific obesity or diabetes risk allele resulted *de novo* before or after archaic/modern human differentiation. With respect to this designation and nomenclature, the ancestral allele refers to the evolutionarily conserved allele dating back to early hominids, while the derived allele refers to a mutated allele that occurred during the course of hominid evolution. Moreover, the ancestral or derived allele may represent either a major allele (greater than 50% allele frequency) or minor allele (lesser than 50% allele frequency) in a race/ethnic group.

### Statistical Analysis

Statistical analysis was performed to determine allele frequencies, linkage-disequilibrium, Hardy-Weinberg equilibrium, and association of alleles with metabolic disease phenotypes, including maternal prepregnancy overweight or category of obesity, HbA1c value, type of diabetes, or large for gestational age (LGA) infants at birth. Outcome data were compiled before patient discharge from the hospital by review of the electronic medical record system. Each HbA1c value was categorized as normal (≤5.6%) or elevated (>5.6%). Diabetes was categorized as preexisting, class A1 gestational diabetes mellitus (A1GDM) defined as requiring dietary treatment, class A2 gestational diabetes mellitus (A2GDM) defined as requiring medical treatment, or diabetes of unknown etiology. A LGA infant was defined as having a birth weight ≥ 90^th^ percentile for gestational age. A power analysis was performed to assess the sample size required for the Chi-square test to detect a range of effect sizes with a 5% level of significance and 80% power after recruiting 245 patients and performing genotype analysis at each loci. The power analysis indicated that 80% power would be attained to determine differences in allele frequency with a total sample size of approximately 300 evaluable patients and that 80% power would be attained to determine association of *NPC1* polymorphisms with metabolic disease phenotypes with a total sample size of approximately 600 evaluable patients. The biometric and genotype data were recorded using statistical package R (Bell Laboratory, Murray Hill, NJ). Pearson’s Chi square test was used to examine differences in allele frequencies for race/ethnic groups. Logistic regression models were used to estimate the odds ratios between specific alleles and metabolic disease phenotypes. An additive genetic model (1/1 versus 1/2 and 1/1 versus 2/2) was used for comparing effects of patients with homozygous ancestral alleles (1/1), heterozygous ancestral and derived alleles (1/2), and homozygous derived alleles (2/2) in race/ethnic groups and total population. This genetic model was used to test for additive effects of the previously reported *NPC1* risk alleles for obesity and diabetes, whereby one risk allele was assessed to increase predisposition by 1x-fold for heterozygous alleles (1/2) and 2x-fold for homozygous alleles (2/2). Although other genetic models exist, the additive genetic model is the most appropriate and commonly used to assess association of genetic determinants with metabolic disease phenotypes (obesity and diabetes). A two-sided P-value < 0.05 was considered to be statistically significant.

## Results

### Demographics of Obstetric Patients in Relation to Race/Ethnic Group

A total of 599 obstetric patients were consented and enrolled for this study. However, due to the relatively low number of Asian-American and African-American patients (a combined total of 32), these patients were excluded to prevent confounding results due to a lack of statistical power. Therefore, a total of 567 obstetric patients that self-identified as non-Hispanic white, Hispanic, or Native American were used for this study (Table 1). The percentage of patients within each race/ethnic group was 26% non-Hispanic white, 62% Hispanic, and 11% Native American. These percentages resemble the proportion for these race/ethnic groups in New Mexico. The results indicated that the mean maternal age for the total population was 27 years (range 18 to 44 years) with a mean maternal prepregnancy BMI of 27kg/m^2^ (range 16 to 58kg/m^2^). More than half of the patients were overweight (25.7%) or obese (30.8%). Furthermore, the obese patients were categorized as either class I obese (17.9%), class II obese (8.5%), or class III obese (4.4%). Non-Hispanic whites and Hispanics had a mean BMI of 27kg/m^2^, while Native Americans had a mean BMI of 29kg/m^2^. An elevated HbA1c (>5.6%) was present in 129 patients (23%), while 46 patients (8%) had either preexisting diabetes, a form of gestational diabetes, or diabetes of unknown etiology.

### Frequency of Alleles and Encoded Residues in Relation to Race/Ethnic Group and Total Population

**Figure 1:**
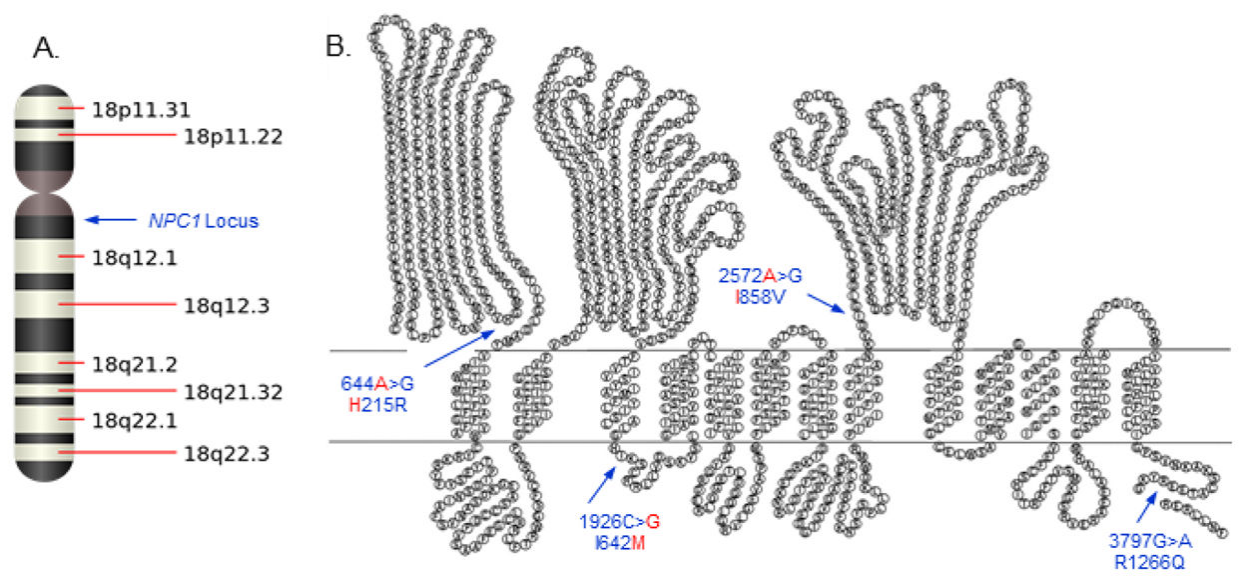
The human *NPC1* chromosomal locus and structure of the NPC1 protein. (A) The human *NPC1* chromosomal locus is positioned on chromosome 18 at cytogenetic band 18q11.2. (B) Structure of the human NPC1 protein. The *NPC1* alleles and encoded NPC1 residues previously reported to be associated with increased risk for morbid-adult obesity (ancestral and major alleles for 644A>G and 2572A>G encoding the H215R and I858V residues, respectively) and type 2 diabetes independent of obesity (ancestral and minor allele for 1926C>G encoding I642M residues) are indicated in red print. An *NPC1* polymorphism and encoded residues (3797G>A encoding R1266Q) serve as a negative control in assessing association of the risk alleles and encoded residues for metabolic disease phenotypes. The structure of the human NPC1 protein used in this figure was adopted from JP Davies and YA Ioannou (2000) J Biol Chem 275:24367–24374 and modified by including the *NPC1* alleles and encoded residues.

The human *NPC1* chromosomal locus and structure of the NPC1 protein are provided (Figure 1). Frequency of alleles and encoded residues for each race/ethnic group and total population are provided (Table 2). The results indicated no significant difference (P = 0.068) in frequency of the 644A>G alleles between race/ethnic groups. In contrast, there was a significant difference (P<0.0001) in frequency of the 1926C>G alleles between race/ethnic groups with non-Hispanic whites having the highest frequency (37%) of the ancestral and minor allele (G) previously reported to be associated with increased risk of type 2 diabetes compared with Hispanics (24%) and Native Americans (11%). Furthermore, there was a significant difference (P<0.0001) in frequency of the 2572A>G alleles between race/ethnic groups with non-Hispanic whites having the highest frequency (51%) of the ancestral and major risk allele (A) previously reported to be associated with increased risk of obesity compared to Hispanics (41%) and Native Americans (26%). There was also a significant difference (P = 0.0003) in frequency of the 3797G>A alleles between race/ethnic groups with non-Hispanic whites having the highest frequency (96%) of the ancestral and major allele (G) compared to Hispanics (87%) and Native Americans (84%). However, neither of the 3797C>A alleles have previously been reported to be associated with increased risk of obesity or diabetes. Further analysis performed using allele and genotype frequencies indicated no significant deviation from Hardy-Weinberg equilibrium within race/ethnic groups. Moreover, the results indicated a significant pairwise linkage-disequilibrium between each of the four loci within race/ethnic groups when using two different standardized measures for linkage-disequilibrium (Table 3). The absolute or scaled pairwise linkage-disequilibrium estimate (D′) between alleles within race/ethnic groups was greater than 0.99, whereby D′ = 1 represents complete linkage-disequilibrium consistent with minimal historical recombination and high pairwise loci inheritance. In contrast, the relative pairwise linkage-disequilibrium (r^2^) between alleles within race/ethnic groups was noticeably lower for some loci comparisons due to the significant differences in allele frequencies. The race/ethnic dependent linkage-disequilibrium of these four *NPC1* polymorphisms may also account for the contrasting associations observed for metabolic disease phenotypes as presented in the next section.

### Association of Alleles and Encoded Residues with Metabolic Disease Phenotypes in Relation to Race/Ethnic Group

Statistical analysis was performed to determine the association of alleles and encoded residues with metabolic disease phenotypes in relation to race/ethnic groups. The results indicated that the ancestral and major allele for 644A>G encoding the H215R residue and the ancestral and major allele for 2572A>G encoding the I858V residue, both previously reported to be associated with increased risk for morbid-adult obesity, were not associated with either overweight or obesity in the race/ethnic groups or total population. Instead, the ancestral and major allele 644A>G encoding the H215R residue trended towards being associated (OR 4.02, 95% CI 0.91–17.8, P = 0.067) with gestational diabetes (A1GDM and A2GDM), while the ancestral and major allele for 2572A>G encoding the I858V residue was associated (OR 4.68, 95% CI 1.23–17.8, P = 0.024) with A1GDM and A2GDM in non-Hispanic whites, but not Hispanics or Native Americans after adjustment for age Table 4.

Moreover, the derived and major allele for 1926C>G encoding the I642M residue was associated (OR 2.10, 95% CI 1.11–3.96, P = 0.022) with maternal prepregnancy overweight (BMI 25.0–29.9kg/m^2^) in non-Hispanic whites, but not in Hispanics or Native Americans after adjustment for age Table 5.

Therefore, the major allele for 1926C>G previously reported to be associated with protection from diabetes and the major allele for 2572A>G previously reported to associated with obesity were differentially associated with maternal prepregnancy overweight and gestational diabetes, respectively, in non-Hispanic whites. Neither of the 3797G>A alleles encoding the R1266Q residues, which were randomly chosen and proposed to serve as negative controls, were associated with increased risk for overweight, obesity, or gestational diabetes in the race/ethnic groups or total population. Moreover, the results indicted no association between any of the *NPC1* polymorphisms with HbA1c levels, gestational age at birth, birth weight, or LGA infants in the race/ethnic groups or total population.

## Discussion

There is an epidemic of obesity and diabetes in the United States that contribute to unfavorable pregnancy outcomes. Many women, regardless of demographics for socioeconomic status, geographical location, and race/ethnic group are vulnerable to overweight or obesity because of limited resources for physical activity, availability of healthy food, employment commitments, and family demands^[22]^. The objectives for this premiere prospective cross-sectional transferability study were to determine allele frequencies for *NPC1* polymorphisms previously reported to be associated with morbid-adult obesity or type 2 diabetes independent of body weight, and to determine whether these *NPC1* polymorphisms are associated with metabolic disease phenotypes in a diverse New Mexican obstetric population.

The results from this study indicated that the allele frequencies for certain *NPC1* polymorphisms (1926C>G encoding the I642M residues, 2572A>G encoding the I858V residues, and 3797G>A encoding the R1266Q residues) were significantly different between non-Hispanic whites, Hispanics, and Native American. Moreover, as anticipated the allele frequencies for each of the four *NPC1* polymorphisms in non-Hispanic whites were similar to the allele frequencies reported for Europeans in the International Hap Map Project^[23]^. Although the allele frequencies for one of the *NPC1* risk alleles previously reported to be associated with morbid-adult obesity (ancestral and major allele for 644A>G encoding the H215R residue) was not significantly different between race/ethnic groups, the allele frequencies of another *NPC1* risk allele associated with morbid-adult obesity (ancestral and major allele for 2572A>G encoding the I858V residue) and type 2 diabetes independent of body weight (ancestral and minor allele for 1926C>G encoding the I642M residue) were highest in non-Hispanic whites (51% and 37%) compared with Hispanics (41% and 24%) and Native Americans (26% and 11%). These results suggest that although Hispanics and Native Americans are at increased risk for these metabolic diseases, it is less likely due to *NPC1* obesity or diabetes risk alleles identified in European populations.

While previous studies have reported that the ancestral and minor allele for 1926C>G encoding the I642M residue is associated with increased risk for type 2 diabetes independent of body weight and that the ancestral and major allele for 2572A>G encoding the I858V residue is associated with increased risk for morbid-adult obesity, our study indicated a differential association of these *NPC1* polymorphisms with these coexistent metabolic diseases. Specifically, the derived and major allele for 1926C>G encoding the I642M residue was associated (OR 2.10, 95% CI 1.11–3.96, P = 0.022) with maternal prepregnancy overweight (BMI 25.0–29.9kg/m^2^) while the ancestral and major allele for 2572A>G encoding the I858V residue was associated (OR 4.68, 95% CI 1.23–17.8, P = 0.024) with gestational diabetes in non-Hispanic whites, but not Hispanics or Native Americans. Consistent with this finding, we have reported that *NPC1* mouse models from different genetic backgrounds (BALB/cJ and C57BL/6J) that share similar decreased *NPC1* gene dosage and loss-of-function mutations are predisposed to either weight gain in the absence of impaired glucose tolerance or impaired glucose tolerance in the absence of weight gain^[24,25]^. Therefore, the *NPC1* polymorphisms at select loci are believed to adversely affect NPC1 protein function and predispose to either weight gain or impaired glucose tolerance depending on race/ethnicity, especially when combined with an obesogenic or diabetogenic environment^[26]^. Moreover, the significant linkage-disequilibrium between each of the four coding non-synonymous *NPC1* polymorphisms may be responsible for cooperatively altering NPC1 protein structure and adversely affecting function that predisposes to different metabolic disease phenotypes.

The strengths of this study are that it represents i) the first time four non-synonymous *NPC1* polymorphisms have been investigated in a multiethnic study and shown to be associated with increased risk for select measures of adiposity and diabetes, ii) the first time these *NPC1* polymorphisms have been investigated in the context of pregnancy, and iii) the first time these *NPC1* polymorphisms have been investigated with assessment of linkage-disequilibrium. The limitations of this study are i) modest sample size and limited power in subgroup analysis, ii) self-reported race/ethnicity which may serve as a suboptimal surrogate of genetic population stratification, and iii) the prospective cross sectional study design prevents causal inference between *NPC1* polymorphisms and metabolic disease phenotypes.

In conclusion, as a result of GWAS identifying obesity and diabetes susceptibility loci, genetic epidemiology has become an emerging field of investigation that will increase our basic knowledge concerning the genetic etiology of common metabolic diseases, including metabolic diseases that adversely affect maternal and newborn health outcomes. We described the allele frequencies of *NPC1* polymorphisms in our New Mexican obstetric population. As hypothesized, our unique population indicated differences in allele frequencies and association with increased risk for metabolic disease phenotypes, including maternal prepregnancy overweight and gestational diabetes in non-Hispanic whites. Information obtained from this study provides novel insight concerning the transferability of a validated obesity and diabetes susceptibility gene which may prove beneficial for future preventative or therapeutic approaches of personalized medicine.

## Figures and Tables

**Table 1:** Demographics of obstetric patients in relation to race/ethnic group

Demographics	Non-Hispanic White (n=150)	Hispanic (n=353)	Native American (n=64)
	Mean (Range)	Mean (Range)	Mean (Range)
Maternal age (years)	29 (18–43)	26 (18–44)	27 (18–41)
Gravida	2 (1–10)	3 (1–12)	3 (1–7)
Height (meters)	1.7 (1.5–1.9)	1.6 (1.3–1.8)	1.6 (1.5–1.8)
Weight (kilograms)	73 (45–135)	69 (41–155)	77 (42–123)
BMI (kilograms/meters^2^)	27 (17–52)	27 (17–58)	29 (16–48)
Diabetes (%)	8.7	10.2	26.6
Pre-existing	1.3	0.6	1.6
A1GDM	2	1.7	6.2
A2GDM	3.3	4.8	9.4
Unknown onset	2	3.1	9.4
HbA1c values (%)	5.4 (4.7–9.0)	5.4 (2.8–7.0)	5.7 (5.1–7.7)
Gestational age at birth (weeks)	39 (27–42)	39 (26–42)	38 (30–42)
Large gestational age infant (%)	4	5.4	6.3

**Abbreviations:** BMI, body mass index; A1GDM, class A1 gestational diabetes mellitus; A2GDM, class 2 gestational diabetes mellitus.

**Table 2:** Frequency of *NPC1* alleles and encoded residues in relation to race/ethnic group and total population

Alleles (Residues)	Non-Hispanic White (n=150)	Hispanic (n=353)	Native American (n=64)	Total Population (n = 567)
644A>G (H215R)				
Major allele (A)^a^	61	70	63	67
Minor allele (G)	39	30	37	33
1926C>G (I642M)				
Major allele (C)	63^d^	76^d^	89^d^	74
Minor allele (G)^b^	37	24	11	26
2572A>G (I858V)				
Major allele (A)^a^	51^d^	41^d^	26^d^	42
Minor allele (G)	49	59	74	58
3797G>A (R1266Q)				
Major allele (G)^c^	96^d^	87^d^	84^d^	89
Minor allele (A)^c^	4	13	16	11

a *NPC1* risk alleles (A) previously reported to be associated with morbid-adult obesity.

b *NPC1* risk allele (G) previously reported to be associated with diabetes independent of body weight.

c *NPC1* allele that has not been reported to be associated with either obesity or diabetes.

d Significant differences (P<0.05) in frequency of the *NPC1* alleles between non-Hispanic whites, Hispanic, and Native Americans using Pearson’s Chi square test.

**Table 3:** Linkage-disequilibrium of *NPC1* alleles and encoded residues in relation to race/ethnic group

Non-Hispanic White		(n=150)		
		R1266Q	I858V	I642M
H215R	D′	0.9968	0.9996	0.9997
	r^2^	0.0657	0.6557	0.9311
	P-value	9.04E-06	< 2.22E-16	< 2.22E-16
I642M	D′	0.9969	0.9996	
	r^2^	0.0705	0.6109	
	P-value	4.24E-06	< 2.22E-16	
I858V	D′	0.9962		
	r^2^	0.043		
	P-value	0.0003		

Hispanic		(n=353)		
		R1266Q	I858V	I642M
H215R	D′	0.9994	0.9996	0.9996
	r^2^	0.3535	0.2967	0.7543
	P-value	< 2.22E-16	< 2.22E-16	< 2.22E-16
I642M	D′	0.9994	0.9993	
	r^2^	0.4683	0.2238	
	P-value	< 2.22E-16	< 2.22E-16	
I858V	D′	0.9989		
	r^2^	0.1049		
	P-value	< 2.22E-16		

Native American		(n=64)		
		R1266Q	I858V	I642M
H215R	D′	0.9992	0.9992	0.9988
	r^2^	0.3378	0.2013	0.2112
	P-value	4.86E-11	3.87E-07	2.01E-07
I642M	D′	0.9991	0.9971	
	r^2^	0.6246	0.0424	
	P-value	< 2.22E-16	0.0198	
I858V	D′	0.9982		
	r^2^	0.0679		
	P-value	0.0031		

D′ = Absolute linkage-disequilibrium estimate; r^2^ = Relative linkage-disequilibrium; P-value = Chi-square P-value for marker independence.

**Table 4:** Association of *NPC1* polymorphisms with increased risk for gestational diabetes

Non-Hispanic Whites			
*NPC1* Allele	OR	95% CI	P-value
644A	4.02	0.91–17.8	0.067
644G	0.26	0.06–1.13	0.072
1926C	0.45	0.17–1.22	0.12
1926G	2.22	0.82–6.03	0.12
2572A	4.68	1.23–17.8	0.024
2572G	0.21	0.056–0.81	0.024
3797G	6.22 × 10^6^	0-∞	0.99
3797A	1.61 × 10^−7^	0-∞	0.99

Hispanics			
*NPC1* Allele	OR	95% CI	P-value
644A	0.60	0.32–1.10	0.099
644G	1.67	0.91–3.10	0.099
1926C	1.09	0.54–2.22	0.81
1926G	0.92	0.45–1.87	0.81
2572A	0.54	0.28–1.07	0.077
2572G	1.84	0.94–3.63	0.077
3797G	1.06	0.45–2.51	0.89
3797A	0.94	0.40–2.23	0.89

Native Americans			
*NPC1* Allele	OR	95% CI	P-value
644A	0.43	0.14–1.22	0.11
644G	2.35	0.82–6.72	0.11
1926C	0.95	0.20–4.55	0.96
1926G	1.04	0.21–4.97	0.96
2572A	2.06	0.49–8.65	0.32
2572G	0.49	0.12–2.04	0.32
3797G	2.49	0.44–14.1	0.30
3797A	0.40	0.07–2.27	0.30

**Table 5:** Association of *NPC1* polymorphisms with increased risk for maternal overweight

Non-Hispanic Whites			
*NPC1* Allele	OR	95% CI	P-value
644A	0.70	0.40–1.22	0.21
644G	1.42	0.82–2.46	0.21
1926C	2.10	1.11–3.96	0.022
1926G	0.47	0.25–0.90	0.022
2572A	0.65	0.36–1.15	0.14
2572G	1.55	0.86–2.77	0.14
3797G	0.95	0.24–3.73	0.94
3797A	1.05	0.26–4.15	0.94

Hispanics			
*NPC1* Allele	OR	95% CI	P-value
644A	0.92	0.60–1.40	0.69
644G	1.09	0.71–1.67	0.69
1926C	0.97	0.62–1.53	0.90
1926G	1.03	0.65–1.62	0.90
2572A	0.89	0.60–1.32	0.56
2572G	1.12	0.76–1.67	0.56
3797G	0.95	0.53–1.72	0.87
3797A	1.05	0.58–1.91	0.87

Native Americans			
*NPC1* Allele	OR	95% CI	P-value
644A	0.69	0.24–1.94	0.48
644G	1.45	0.5–4.10	0.48
1926C	2.66	0.34–21.1	0.35
1926G	0.37	0.05–2.98	0.35
2572A	0.94	0.23–3.90	0.94
2572G	1.06	0.26–4.37	0.94
3797G	0.72	0.21–2.37	0.59
3797A	1.39	0.42–4.57	0.59
